# Ambiguous at the second sight: Mixed facial expressions trigger late electrophysiological responses linked to lower social impressions

**DOI:** 10.3758/s13415-020-00778-5

**Published:** 2020-03-12

**Authors:** Olga Katarzyna Kaminska, Mikołaj Magnuski, Michał Olszanowski, Mateusz Gola, Aneta Brzezicka, Piotr Winkielman

**Affiliations:** 1 University of Social Sciences and Humanities, Warsaw, Poland; 2 Institute for Neural Computation, Swartz Center for Computational Neuroscience, University of California, San Diego, La Jolla, CA USA; 3 Institute of Psychology, Polish Academy of Sciences, Warsaw, Poland; 4 Psychology Department, University of California, San Diego, La Jolla, CA USA; 5 Department of Neurosurgery, Cedars-Sinai Medical Center, Los Angeles, CA USA

**Keywords:** Processing fluency, Trustworthiness, EEG/ERP, Face processing

## Abstract

Social interactions require quick perception, interpretation, and categorization of faces, with facial features offering cues to emotions, intentions, and traits. Importantly, reactions to faces depend not only on their features but also on their processing fluency, with disfluent faces suffering social devaluation. The current research used electrophysiological (EEG) and behavioral measures to explore at what processing stage and under what conditions emotional ambiguity is detected in the brain and how it influences trustworthiness judgments. Participants viewed male and female faces ranging from pure anger, through mixed expressions, to pure happiness. They categorized each face along the experimental dimension (happy vs. angry) or a control dimension (gender). In the emotion-categorization condition, mixed (ambiguous) expressions were classified relatively slower, and their trustworthiness was rated relatively lower. EEG analyses revealed that early brain responses are independent of the categorization condition, with pure faces evoking larger P1/N1 responses than mixed expressions. Some late (728- 880 ms) brain responses from central-parietal sites also were independent of the categorization condition and presumably reflect familiarity of the emotion categories, with pure expressions evoking larger central-parietal LPP amplitude than mixed expressions. Interestingly, other late responses were sensitive to both expressive features and categorization task, with ambiguous faces evoking a larger LPP amplitude in frontal-medial sites around 560-660 ms but only in the emotion categorization task. Critically, these late responses from the frontal-medial cluster correlated with the reduction in trustworthiness judgments. Overall, the results suggest that ambiguity detection involves late, top-down processes and that it influences important social impressions.

**Electronic supplementary material:**

The online version of this article (10.3758/s13415-020-00778-5) contains supplementary material, which is available to authorized users.

## Introduction

Social interactions often require quick perception, interpretation, and categorization of faces (Todorov, 2017). Facial features offer cues to a target’s emotional states, personality, and behavioral intentions, as documented by decades of work on facial displays (Knutson, 1996; Russel, Bachorowski, & Fernadez-Doz, 2003). Facial features also allow people to form rapid judgments about important social traits, including trustworthiness—a major element in relationships, trade, and politics (Rezlescu, Duchaine, Olivola, & Chater, 2012; Todorov, Pakrashi, & Oosterhof, 2009; Wojciszke, Bazinska, & Jaworski, 1998). Interestingly, the processes involved in emotion perception and impression formation seem tightly intertwined (Ambady & Skowronski, 2008). For example, people judge a face as more trustworthy when it has features resembling smiles and less trustworthy when the face resembles anger (Oosterhof & Todorov, 2008).

Most of the existing research has focused on understanding the psychological and neural underpinnings of facial feature processing in emotion perception and impression formation. However, people’s evaluative judgments and affective responses depend not only on features but also on the fluency (ease or difficulty) with which such features are processed. For example, when the task requires categorizing faces on some dimension, a face ambiguous on that dimension becomes disfluent, which lowers its evaluation (Halberstadt & Winkielman, 2014; Winkielman, Olszanowski, & Gola, 2015; Olszanowski, Kaminska, & Winkielman, 2018; Owen, Halberstadt, Carr, & Winkielman, 2016). The negative reaction caused by disfluency is sometimes strong enough to override the benefits of positive stimulus features. For example, objectively happier but disfluent faces are sometimes rated as *less* trustworthy than objectively angrier but fluent faces (Winkielman et al., 2015). This presumably occurs because disfluency triggers negative affect, which perceivers then use when judging the effort-inducing stimulus.

Despite several recent demonstrations of such effects on evaluations, their underlying mechanisms are poorly understood. Specifically, we do not know when (at what processing stage) ambiguity is detected, how ambiguity detection depends on task requirements, and what kind of ambiguity influences evaluative judgments. In the current article, we used behavioral and physiological (EEG) measures to explore these questions by examining the temporal dynamics of brain processing of emotional faces during emotional perception, categorization, and social judgments. Our investigation helps us to better understand the processing of facial expression and the formation of first impressions, including socially important trustworthiness judgments. Understanding the role of ambiguity in processing facial expressions is important given that in the real world, faces are “inherently ambiguous” and often contain features of multiple emotions (Aviezer et al., 2008; Hassin, Aviezer, & Bentin, 2013). This investigation also helps us to extend our understanding of the mechanisms underlying ambiguity processing more generally, as similar behavioral effects in evaluation have been obtained with a variety of facial and nonfacial stimuli (Carr et al., 2017a; Carr et al., 2017b; Sun et al., 2017; Winkielman, Halberstadt, Fazendeiro, & Catty, 2006). Before we describe the current study, we offer more background on the concept of processing fluency and the EEG measures of face processing in the brain.

### Processing Fluency in Perception and Categorization and its Impact on Evaluation

Processing fluency is the effort of perceptual and conceptual mental operations and is typically indexed using reaction times (Jacoby, Kelley, & Dywan, 1989), subjective measures of ease (Oppenheimer, 2008), and electrophysiological measures, such as EEG (Nessler et al., 2005; Trujillo, Jankowitsch, & Langlois, 2014). Previous studies demonstrated two major classes of fluency determinants. First, fluency depends on low-level physical stimulus characteristics, for example, clarity or contrast, or basic stimulus features, such as simplicity, symmetry, regularity, or a simple match with stored patterns. These factors influence “*perceptual fluency.*” Second, fluency also reflects conceptual aspects of stimulus processing, such as categorization ease. Importantly, such “*conceptual fluency*” depends on the current task. For example, the same multidimensional stimulus might be conceptually fluent or disfluent, depending on whether the categorization task draws on a stimulus dimension that is easy or difficult to categorize (Winkielman et al., 2015). As mentioned, fluency matters for evaluation, with easier processing generally enhancing favorability ratings and eliciting positive affective responses (Schwarz, 2007; Winkielman & Cacioppo, 2001; Winkielman, Schwarz, Fazendeiro, & Reber, 2003). These effects have been demonstrated in the face processing domain, using dimensions, such as gender, ethnicity, or emotion (Halberstadt et al., 2014). For example, in previous studies that inspired the current research, participants were shown male and female faces ranging from pure emotion (anger or happiness) to mixed emotion (blended anger and happiness). Participants needed to evaluate these faces on a social dimension (attractiveness, trust). Before their judgments, participants were asked to categorize faces based on either an emotional dimension or a control dimension (e.g., gender). Results showed that ambiguous, mixed emotion stimuli (angry-happy blends) were evaluated less positively in comparison to pure emotion stimuli, especially when participants first needed to categorize the stimulus on the emotion dimension. In addition, physiological measures of affective responses (facial EMG) showed decreased smiling to disfluent stimuli. Finally, categorization fluency (RTs) statistically mediated the impact of stimulus ambiguity (mixedness) on evaluative ratings (Olszanowski et al., 2018; Winkielman, Olszanowski, & Gola, 2015). Yet, as mentioned, the psychological and neural mechanisms underlying these effects are poorly understood. Therefore, it is necessary to examine the role of different stages and components of facial processing, their neural signatures, and their links with social impressions.

### Stages of Face Processing and Social Evaluation

Processing of emotion and faces is a complex, gradual, and interactive process (Adolphs, 2002; Cunningham & Zelazo, 2007). However, for the current purposes, it can be roughly divided into perceptual (early) vs. conceptual (late) conceptual stages (Bruce & Young, 2012; Haxby, Hoffman & Gobbini, 2002). Early stages involve construction of the representation from the perceptually available facial features and matching it to existing templates. In contrast, later stages involve linking the constructed perceptual representation with existing knowledge about emotional categories and social knowledge. This early-late distinction is important for understanding what happens when a perceiver encounters a display of mixed emotion, such as a mix of happiness and anger. At the earlier, perceptual stages, a mixed expression represents a poorer match to a preexisting feature template than a relatively pure, or more “prototypical” expression (Russell et al., 2003). At the later stages, the perceiver must resolve a conflict between the perceived mix of facial features and the activated emotion categories, especially when the current task requires the perceiver to do so. Resolution of this conflict takes cognitive effort, recruiting cognitive, motivational, and affective resources (Sun et al., 2017; Willadsen-Jensen & Ito, 2006). The resolution of the conflict then allows the perceiver to assign the faces into some emotion category that can then support social judgment.

### ERP Measures of Face Processing

To better understand the dynamics of face perception, categorization, and evaluation, researchers have often relied on EEG measures, especially event-related brain potentials (ERPs). Such measures allow for precise assessment of temporal dynamics (Eimer & Holmes, 2007) and provide information about the mental effort involved in such processing (Sun et al., 2017). Accordingly, we use ERP measures to explore early and late effects, as a function of the physical features of the faces and the impact of the categorization task. We briefly review three ERPs of interest for the current research: P1, N170, and LPP (late positive potential).

One of the earliest ERP responses to facial stimuli occurs around 100 ms after stimulus onset and is known as the P1 potential. It appears as a positive amplitude deflection at posterior electrode sites around 90-100 ms (Dering, Martin, Moro, Pegna, & Thierry, 2011). The P1 does not reflect activation of a complete face representation but instead reflects image properties that resemble the face, such as roundness, specific color distribution, spatial frequency, and contrast maps (for review see Luck & Kappenman, 2012). Interestingly, there is some evidence that the P1 is sensitive to the type of facial expression presented, at least under focal attention. Presumably, this reflects processing of low-level features that match a template for a specific expression, such as anger, fear, or happiness (Holmes, Vuilleumier, & Eimer, 2003). Note that concurrent to the P1, the fronto-central negativity, the N1 potential occurs around 100–150 ms (Pourtois, Thut, Grave de Peralta, Michel, & Vuilleumier, 2005). The N1 and P1 are presumably generated by the same dipolar source in the brain and thus reflect the same processes (Rossion et al., 1999a, b).

Another ERP, the N170, is linked to the structural encoding of faces (Eimer & Holmes, 2007). It peaks approximately 150-190 ms after stimulus onset over temporo-occipital electrode sites (Rossion & Jacques, 2008). The N170 is sensitive to differences in emotional display, with greater (i.e., more negative) amplitude to angry than happy faces (Krombholz, Schaefer, & Boucsein, 2007). Interestingly, the N170 also is sensitive to fluency of perceived stimuli. For example, N170 response is reduced to more familiar faces (Caharel, Courtay, Bernard, Lalonde, & Rebaï, 2005), faces resembling a global prototype, such as averaged faces obtained by blending many individual faces (Trujillo et al., 2014), and faces displayed in the canonical, upright orientation compared with rotated faces (Rossion et al., 1999a, b; Magnuski & Gola, 2013).

The late positive potential ERP component, the LPP, appears 400-800 ms poststimulus onset and reflects more complex processes associated with memory, categorization, and evaluation of the affective and motivational significance of the stimulus for the perceiver (Friedman & Johnson, 2000; Frenkel & Bar-Haim, 2011; Schacht & Sommer, 2009; Sun et al., 2017). Critically, previous work has distinguished the timing and presumed brain sources of different psychological processes generating distinct types of LPP response.

LPP responses at central-parietal sites are related to two sets of processes. One is a sense of familiarity, which is believed to be relatively contextually independent (Rugg et al., 1998). The other relates to the emotional intensity of a stimulus—positive or negative valence stimuli both elicit relatively larger (i.e., more positive) LPP amplitudes than neutral stimuli (Duval, Moser, Huppert, & Simons, 2013; Hajcak, MacNamara, & Olvet, 2010; Cuthbert, Schupp, Bradley, Birbaumer, & Lang, 2000). Some LPP responses from central-parietal sites are relatively independent of aspects of the presentation context (Pastor et al., 2008), although a recent review suggests top-down goals and task requirements play an important role, especially when they change the motivational significance of the stimulus (Hajcak & Foti, in press).

Interestingly, research suggests that some components of the LPP are sensitive to task requirements and may reflect processes related to effort and decision-making. Specifically, more frontal LPPs differentiate levels of ambiguity and depend on the choice requirements, rather than passive perception of ambiguous stimuli (Calvo, Marrero, Beltran, 2013; O'Connell et al., 2012; Kelly and O'Connell, 2013; Murphy et al., 2015). Recently Sun et al. (2017) have shown greater LPP responses to ambiguous stimuli, including mixed facial expressions, but only when participants had to make a categorization choice. In their study, which used a similar paradigm to our investigation, stimuli varied on two dimensions, and participants made classification decisions that were either related or unrelated to the dimensions on which the stimuli were ambiguous. Greater (more positive) LPP responses were found to stimuli that were ambiguous but only when they needed to be judged on the ambiguous dimension. Using a combination of EEG and fMRI methods, Sun et al. (2017) interpreted this response as reflecting greater processing effort, perhaps due to greater engagement of brain areas involved in conflict resolution. We will return to the LPP interpretation issues in the *Discussion*.

### Current Study

Based on the background presented above, we can now more precisely articulate the hypotheses of the current study. As a reminder, our primary goal was to understand the dynamics of processing of pure and mixed (ambiguous) emotional expressions and how that relates to social evaluations, such as trustworthiness. Furthermore, we wanted to understand how such processing depends on stimulus characteristics and categorization conditions.

**Fig. 1:**
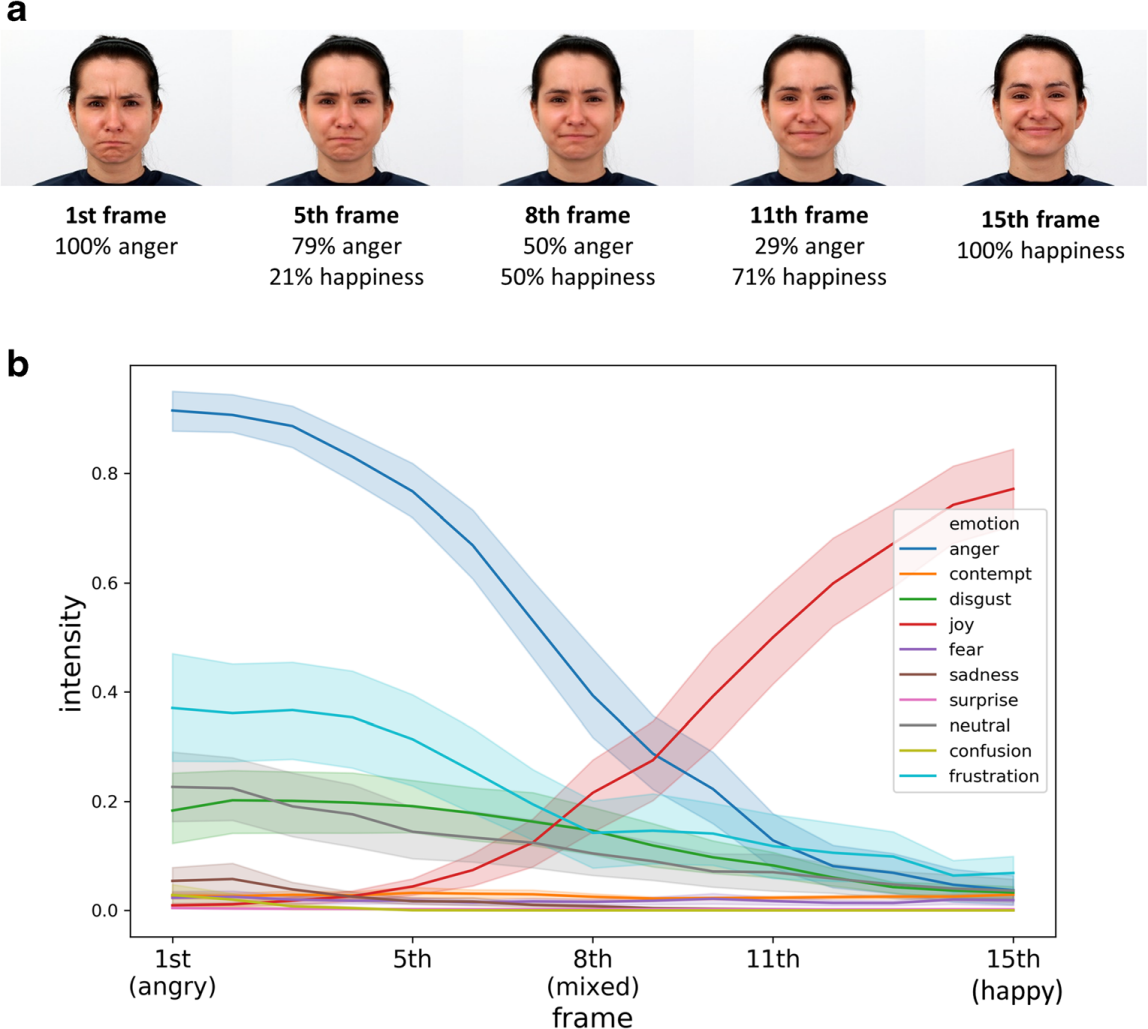
Panel A: Examples of stimuli used in the current experiment. Expressions varied from angry to happy. On the x axis is the percentage of anger and happiness mixed within each frame. Panel B: Results from Computer Expression Recognition Toolbox (CERT) analyses of all experimental stimuli indicating intensity of 10 different expressions

**Fig. 2:**
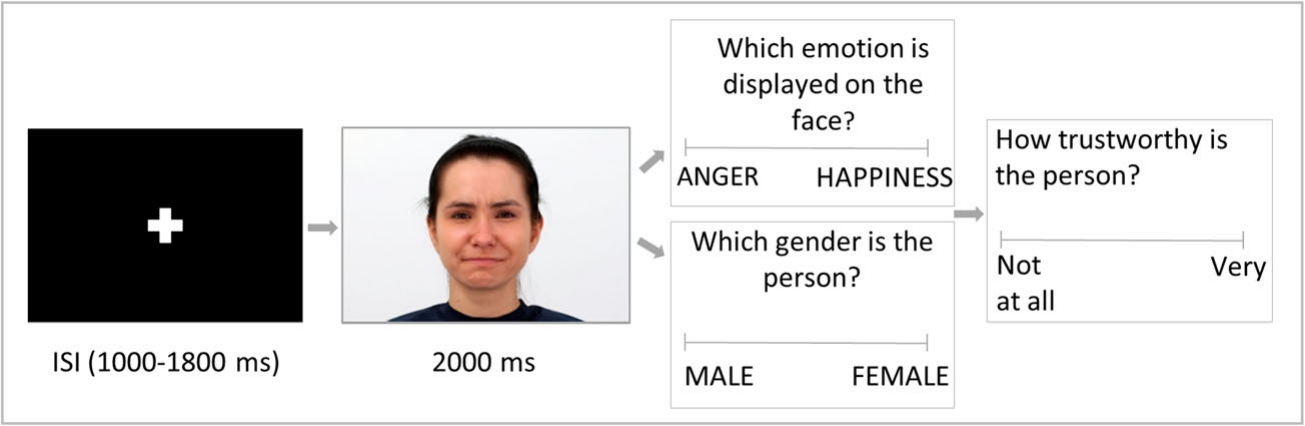
Example trial from the behavioral task

To do so, we designed the following task based on previous research in our lab (Olszanowski et al., 2018; Winkielman et al., 2015). Participants were asked to view male and female faces with emotional displays ranging from clear happiness through mixed emotions (happiness mixed with anger) to clear anger (Fig. 1). Participants categorized these faces along the dimension of either gender (control condition) or emotion (experimental condition) (Fig. 2). Because the faces gradually ranged from happy to angry, we expected that the middle stimuli would be difficult to categorize on the emotion dimension. Gender dimension was bipolar (male vs. female) and never mixed, so we expected that such stimuli would be always easy to categorize. After categorizing the face, participants evaluated it on a trustworthiness scale. During the entire procedure, we recorded EEG as a measure of processing dynamics.

On the behavioral level, we expected to replicate previous results (Winkielman, Olszanowski, & Gola, 2015). In terms of fluency, mixed expressions should take longer to categorize, compared with clear expressions, but only when participants categorize faces on the emotion dimension (experimental condition). In terms of trust ratings, mixed expressions should receive relatively lower trust evaluations but only during emotion categorization. This is because disfluency (effort) associated with resolving ambiguity is negatively tinged and spills over to stimulus evaluation (Winkielman et al., 2003).

On EEG measures, we expected to observe differences at early and later stages of face processing as a function of stimulus characteristics and participants’ task. As discussed, the early responses (P1/N1, N170) to emotional displays should primarily reflect their match to their respective emotion templates, with pure (clear happiness, and clear anger) facial expressions evoking larger amplitudes of their relevant component. Our expectation for late responses (LPP) was more nuanced. As mentioned, the literature on decision effort in emotion categorization predicts modulation of the ERP amplitude by the categorization task (Sun et al., 2017). Specifically, in the emotion categorization condition, LPP responses to mixed expressions should be *larger* (more positive), due to disfluency (effort) involved in disambiguation of such displays. However, other literature suggests that the LPP also reflect emotional intensity and familiarity of facial expressions, relatively independent of context (Pastor et al., 2008; Rugg et al., 1998). Given that mixed displays are less familiar, less intense, and less representative of prototypical emotion categories, they may evoke smaller (less positive) responses than pure expressions on LPP components sensitive to those processes. We return to these nuances in the discussion.

## Method

### Subjects

Thirty-one healthy participants with normal or corrected-to-normal vision took part in the study (11 males, age range 19-29 years, M = 22.2, standard deviation [SD] = 2.5). The Ethical Review Board at the SWPS University of Social Sciences and Humanities approved the study and all participants (students or recent graduates from SWPS University) gave informed consent. Participation in the study was voluntary and assignment to the groups was randomized. Two subjects were removed from behavioral data analysis due to outlier reaction times (2 SD above the group’s mean). Two subjects were discarded from EEG analysis, due to the low EEG signal quality (excessive artifacts).

### Stimuli and Experimental Procedure

The stimuli used in this study came from the Warsaw Set of Emotional Facial Expression Pictures (Olszanowski et al., 2015). From this set, we selected 16 individuals (8 females and 8 males) who provided 2 expressions each (anger and happiness). Mixed expression faces were constructed using FantaMorph 5 software by combining (in different proportions) two source images of “pure” expressions. Transitions within 13 steps of each expressions pair were delineated using over 100 facial landmarks, resulting in 15 pictures from each poser (model). To limit the total number of presented stimuli, we selected five frames (1^st –^ anger, 5^th,^ 8^th^, 11^th^ – mixed to a different degree and 15^th^ – pure happiness; Fig. 1A). This resulted in 90 different total pictures. To “objectively” test the emotional intensity and emotional classification of these stimuli, we analyzed all the pictures with the Computer Expression Recognition Toolbox - CERT (Bartlett et al., 2008). The results are shown in Fig. 1B (the underlying pictures and data are available on request). This analysis showed that pictures of pure (100%) expressions of anger and happiness yielded the maximum intensity values of intended emotion (anger, joy) and that pictures of mixed expressions (50/50) yielded lower intensity levels of pure expression and did not reach high-intensity level for other tested emotions (e.g., contempt, disgust, sadness, etc.).

The experimental procedure was programmed using the Inquisit 3 software, which interfaced with software for EEG data collection. Participants were randomly assigned to one of the two conditions: control condition (gender categorization) and experimental condition (emotion categorization). The presentation of stimulus trials was grouped in two identical blocks of 240 trials (each picture was presented 3 times). Every trial consisted of the following four events. First, a fixation cross appeared for a randomized duration in range of 1,000 to 1,800 ms in 200-ms intervals. Second, a target face stimulus was presented for 2,000 ms. Third, a categorization task, where participants indicated the appropriate category by clicking on a virtual button located approximately 50 pixels above and below the center of the computer screen (while the face was centrally located). Fourth, for each face, participants responded to the question “Is this person trustworthy?” by moving a 100-point slider (with anchors from “not at all” to “very”). See Fig. 2 for a schematic representation of an experimental trial.

#### EEG Acquisition

The EEG signal was acquired using 64 channels EGI Geodesics GES 300 amplifier. The EEG was sampled at 250 Hz, using the NetStation software. No online filters were used. Data was referenced to Cz electrode. The impedances were kept below 50 kΩ.

#### EEG Preprocessing

We ran the EEG data analysis using the EEGLAB toolbox (12.0.1) (Delorme & Makeig, 2004), Fieldtrip (Oostenveld, Fries, Maris, & Schoffelen, 2011), and custom Matlab scripts (Matlab R2013a, MathWorks). First, we applied 1- to 45-Hz band pass FIR filter, and to remove the remaining 50-Hz noise in the signal, we applied an additional Clean Line filter (Mitra & Bokil, 2007). To ensure good quality of Independent Component Analysis (ICA), we followed the practice of using the maximum amount of data for the decomposition. Therefore, ICA was not limited to the actual segments that were later used in analysis but was performed on full data after exclusion of major artifacts via visual inspection (such as large signal distortions due to movement, periods of strong myogenic artifacts or other substantial nonrepeatable events). The quality of Independent Components (ICs) was assessed for each subject visually by examining the EEGLAB component properties dialog. At this stage, the weights of the ICs were stored in a matrix form for further usage.

Next, we went back to the original, raw data and applied a previously used filtering setup. We epoched the data into 1,500-ms segments (from 500 ms before the stimulus onset up to 1,000 ms after). After that, we applied previously obtained ICA weight matrices to this signal. This allowed us to use ICs extracted from full data on epoch-limited segments, therefore, enhancing the quality of ICA-driven artifact rejection. To objectively remove artifact ICs, we used CORRMAP EEGLAB plugin (Viola et al., 2009): first, we defined the IC template for blink, eye movement, and cardiac artifacts and then CORRMAP identified ICs with high correlation (above threshold of r = 0.87) with the template for each participant. Then, we conducted the final visual inspection of the ICA-cleaned epoched data and rejected any remaining noise and artifacts. These preprocessing steps led on average to 413.4 trials remaining in the emotion condition (86.13% of all trials, SD = 37.3 trials; 7.78%) and 378.7 trials in the gender condition (78.90% of all trials, SD = 63.2; 13.16%). The difference between the number of trials remaining in each condition was not significant (t = 1.71, *p* = 0.10). Finally, we re-referenced the data to the average from all 64 electrodes and baselined the signal with respect to a period of 500 ms before stimulus presentation (−500 ms up to 0 ms).

### EEG Analysis

All EEG analyses were conducted using nonparametric cluster-based permutation tests (Maris and Oostenveld, 2007). The reader unfamiliar with these analyses will find additional information in *Supplementary Materials*.

#### Application of cluster-based analyses to the current data

To assess the overall effect of ambiguity (pure vs. mix), we compared the ERPs obtained from the middle frame to ERPs obtained from the combined happy and angry frames. The cluster-based permutation test was used on the search space consisting of all channels and all-time samples using repeated measures *t*-test as the test statistic and 1,000 permutations. Cluster membership threshold was set to *t* > 2.052 for positive effects and *t* < −2.052 for negative effects. This *t*-value corresponds to *p* = 0.05 for a t-test with 27 degrees of freedom (our repeated measures case).

To quantify the interaction effect, we performed a two-step analysis, popular when using cluster-based tests to test for interactions. In the first step, we computed the contrast between mix and pure frame ERPs within each participant using a *t*-test. Because we expected this interaction effect to appear late and be located frontally, we restricted the search space to a time window of 400-1,000 ms and frontal channels. This step resulted in one channel by time t statistic map per participant—each representing the within-subject pure vs. mix contrast. Then, in the second step, we used cluster-based approach to test for inequality in these t-value maps depending on categorization condition using independent *t*-test as the test statistic and 1,000 permutations. Cluster membership threshold was set to *t* > 2.056 for positive effects and *t* < −2.056 for negative effects. This t-value corresponds to *p* = 0.05 for a *t*-test with 26 degrees of freedom (our independent measures case).

## Results

### Behavioral Data

Data were analyzed with a 2 (categorization: emotion vs. gender) × 5 (emotion: anger to happiness) mixed-model ANOVA. According to our hypothesis, in the emotion categorization condition, the fluency (RT) curve should assume an inverse U-shape, whereas the trust evaluation curve should follow a U-shape. Accordingly, we tested for the presence of quadratic contrasts and their interaction with classification condition.

#### Reaction times

**Fig. 3:**
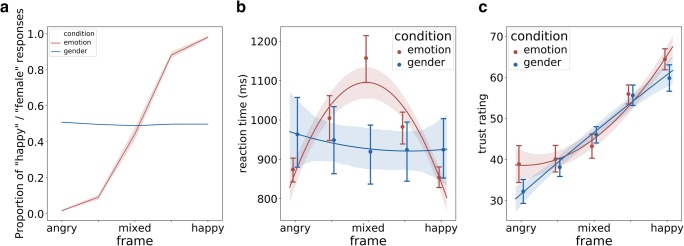
Panel A: Proportion of categorizations for the emotion and gender conditions for the pure and mixed expressions. Panel B: Reaction times for the emotion and gender categorization conditions. Panel C: Trustworthiness judgments for the emotion and gender categorization conditions. Error bars represent standard error of the mean

Categorization times for mixed expressions depended on the experimental condition. In the emotion condition, participants took longer to categorize mixed expressions (5^th^ frame: *M* = 1,005 ms, *SD* = 249; 8^th^ frame: *M* = 1,157 ms, *SD* = 246; 11^th^ frame: *M* = 983 ms, *SD* = 166) than pure expressions (Anger – 1^st^ frame: *M* = 874 ms, *SD* = 135; Happiness – 15^th^ frame: *M* = 854 ms, *SD* = 114). This resulted in significant quadratic (inverse U –shape) contrast, *F*(1, 15) = 45.13, *p* < 0.001, η^2^ = 0.751. In the gender condition, categorization time did not differ across morph frames (Anger – 1^st^ frame: *M* = 964 ms, *SD* = 343, 5^th^ frame: *M* = 949 ms, *SD* = 319; 8^th^ frame: *M* = 919 ms, *SD* = 283; 11^th^ frame: *M* = 924 ms, *SD* = 284; Happiness – 15^th^ frame: *M* = 925 ms, *SD* = 309). Comparing these two patterns yielded a significant interaction with quadratic (inverse U-shape) contrast: *F*(1, 27) = 32.61, *p* < 0.001, η^2^ = 0.547 (Fig. 3B).

#### Trustworthiness

In both conditions, judgments of trustworthiness increased with the percentage of happiness contributing to the expression. The linear contrast was significant for the gender categorization condition, *F*(1, 12) = 39.14, *p* < 0.001, η^2^ = 0.765, (1^st^ frame: *M* = 32.20, *SD* = 10.34; 5^th^ frame: *M* = 38.11, *SD* = 7.98; 8^th^ frame: *M* = 46.017, *SD* = 7.76; 11^th^ frame: *M* = 55.61, *SD* = 9.96; 15^th^ frame: *M* = 59.83, *SD* = 12.05). It also was significant for the emotion categorization condition, *F*(1, 15) = 31.33, *p* < 0.001, η^2^ = 0.676, (1^st^ frame: *M* = 38.88, *SD* = 17.85; 5^th^ frame: *M* = 40.07, *SD* = 13.39; 8^th^ frame: *M* = 43.20, *SD* = 11.87; 11^th^ frame: *M* = 55.96, *SD* = 9.60; 15^th^ frame: *M* = 64.45, *SD* = 10.15). Importantly, we also observed a significant quadratic contrast for trustworthiness ratings in the emotion categorization condition, *F*(1, 15) = 6.61, *p* = 0.021, η^2^ = 0.306, but not in the gender categorization condition. Altogether, comparing both conditions resulted in significant quadratic interaction *F*(1, 27) = 5.74, *p* = 0.024, η^2^ = 0.175 with higher quadratic contrast (U-shaped dependency) in the emotion categorization condition. The quadratic contrast in the emotion categorization condition reflects relatively lower trust evaluation for mixed frames (Fig. 3C – right graph). This shows that in the gender condition, the trustworthiness ratings increase due to a greater amount of happiness in the facial stimulus, while in the emotion categorization condition, trustworthiness changes not only as a function of transition from anger to happiness but also due to increased mixedness/ambiguity of the middle frames, resulting in a quadratic contrast.

### EEG Results

As discussed, our theoretical focus was the difference between brain responses to pure versus ambiguous expressions. For this reason, and to simplify the EEG analyses, we created two categories of stimuli: (I) “pure” emotional expressions (100% happiness and 100% anger expressions combined) and (II) “mixed” expressions (middle frames). We first looked at the overall mixedness effect—a comparison between pure vs mixed expressions, which we then followed with an analysis of the mixedness by categorization condition interaction. The clusters reported below were obtained using the cluster-based permutation test (for details see the *EEG Analysis* subsection in the *Methods* and also in *Supplementary Materials*). As discussed in methods, the analyses looking at mixedness of stimuli were performed on the full channel x time ERP space. The mixedness x categorization condition interaction was expected to appear frontally and late. Accordingly, these analyses were conducted on a more restricted search space (frontal channels and time window of 400-1,000 ms). Below we report the results by grouping them into early and late effects.

#### Early Effects

**Fig. 4:**
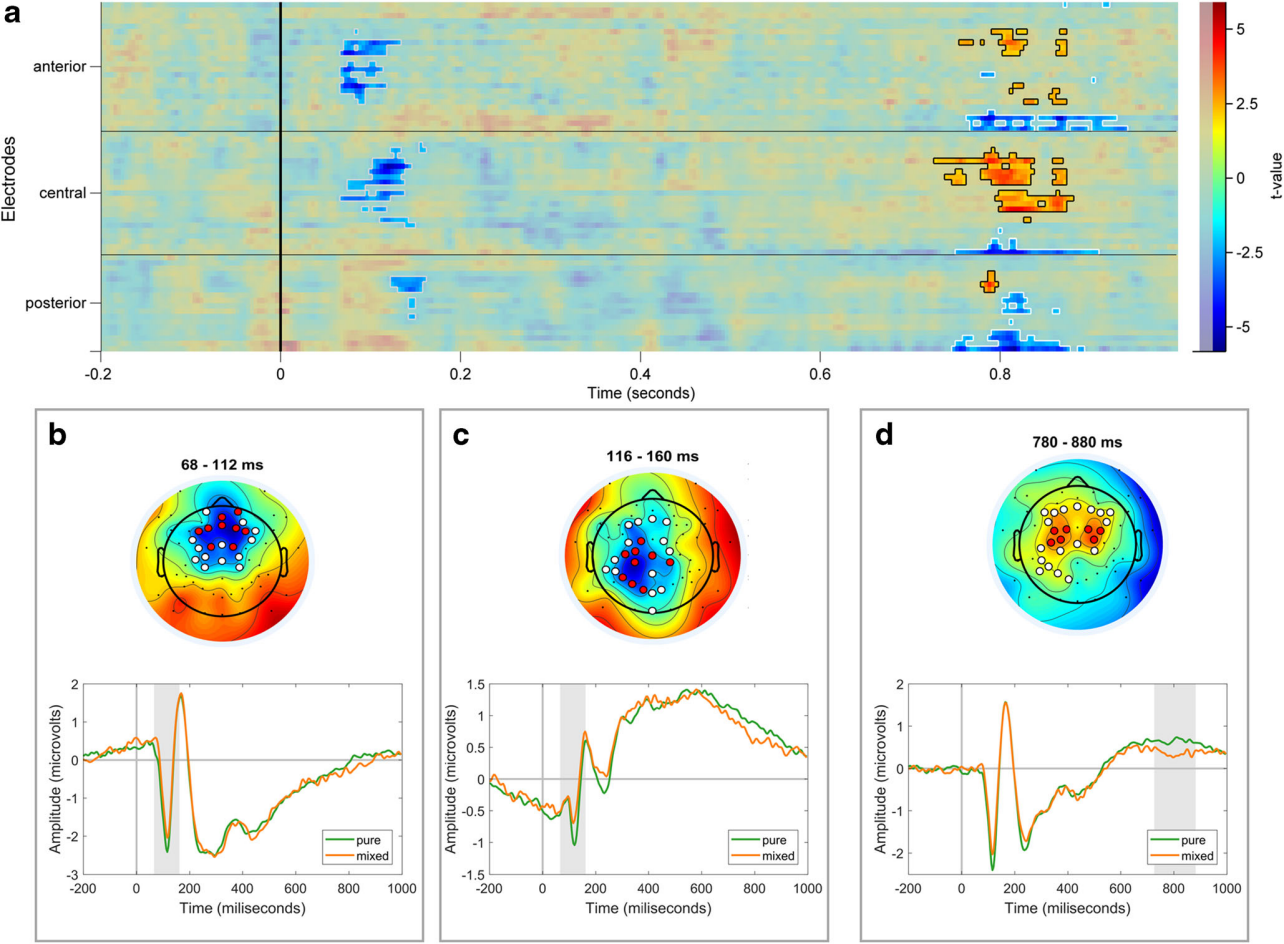
EEG results for pure vs mix comparison (Panels A, B, C, D). The A panel represents the cluster plot- a heatmap of effects in channel by time space. Colors represent the strength and direction of the pure versus mix comparison (t-values, blue – greater amplitude for the mix category; red – greater amplitude for the pure category). Significant clusters are marked with contour lines and more vivid colors. Panels B, C and D show the topography and ERP time course of the early negative cluster (Panel B and C) and the late positive cluster (Panel D). The ERPs are obtained by averaging channels that participate in the cluster for at least 50% of the specified time range (displayed above the topography). These channels are marked in red on the topography plot, the remaining cluster channels (for the specified time range) are marked in white. The topography represents the mean t-values for representative cluster time windows

During early periods of sensory processing, the mixedness analysis revealed a negative (blue) cluster extending from 68 ms to 160 ms (*p* = 0.03; Fig. 4A for the whole cluster extent and Panels B and C for representative topography and time course). Note that the difference within this negative cluster co-occurs with a large negative peak at frontal-midline topography (N1), with more negative amplitude for pure than for mixed frames. Although we found a robust N170 response in the grand average ERP, we did not detect any significant differences between pure and mixed emotion expressions, nor any interaction effects within the N170 spatio-temporal window. In short, as expected, the early ERP components appeared to reflect the process of matching perceived facial expressions to emotion templates.

#### Late Effects

The mixedness analysis revealed one late positive (red) cluster, ranging from 728 ms to 880 ms (*p* = 0.03; Fig. 4A and D) and one late negative (blue) cluster, ranging from 748 ms to 880 ms (*p* = 0.018). The late positive cluster occurs at central electrodes with more positive values for pure than for mixed expressions, while the late negative cluster seems to originate from the same dipolar source. As we elaborate in the discussion, this late component probably reflects LPP and is related to stimulus intensity and familiarity.

**Fig. 5:**
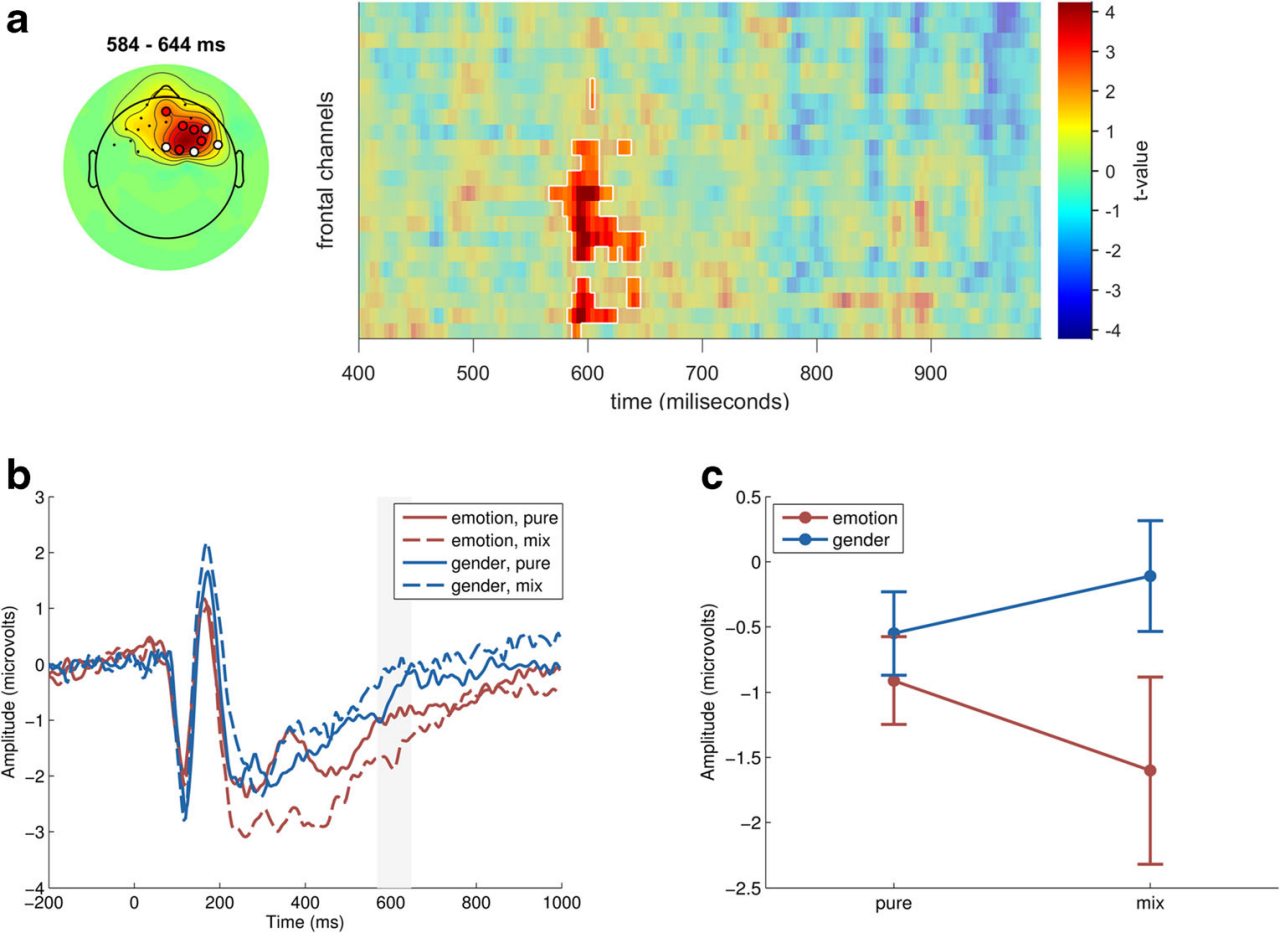
Stimulus type x categorization condition interaction effect (Panels A, B, C). A) Topography and cluster plot, using the same visualization method as in Fig. 4B) ERP plot of the interaction effect, using the same visualization method as in Fig. 4C) Line plot of the interaction effect for the averaged cluster time window. The error bars represent 95% confidence interval

The interaction analysis revealed a component where the mixedness effect was dependent on the categorization task, as reflected in a significant interaction between mixedness level and categorization task. Interestingly, this effect occurred a bit earlier than the late mixedness effect—within a 566 ms to 644 ms time window—and had a frontal midline topography (*p* = 0.034). As shown in Fig. 5A, B, and C, this interaction reflects greater amplitude to mixed faces in the emotion categorization condition as compared to the control condition (gender categorization). As we elaborate in the discussion, this task-dependent late component may reflect decision effort.

#### Late Positive Interaction Cluster Correlates with Behavioral Effects

As the final step, we examined the link between brain responses and two behavioral measures: trustworthiness and categorization RT. To this end, we computed trust rating “bend” as the difference between observed trust rating for the mixed emotion frame and trust rating expected for the same frame based on linear interpolation obtained from two edge frames (pure happiness and pure anger). This approach is conceptually identical to calculating quadratic contrast for trust ratings (this quadratic relationship can be seen in Fig. 3C). Trust rating bend was then correlated with single-subject t-value contrasts (mix vs pure condition) from the interaction cluster (566 ms to 644 ms at frontal midline topography). T-values were used because the interaction cluster was obtained in a two-step approach where single-subject t-value maps are contrasted between conditions (see details in *EEG Analysis* methods subsection).

**Fig. 6:**
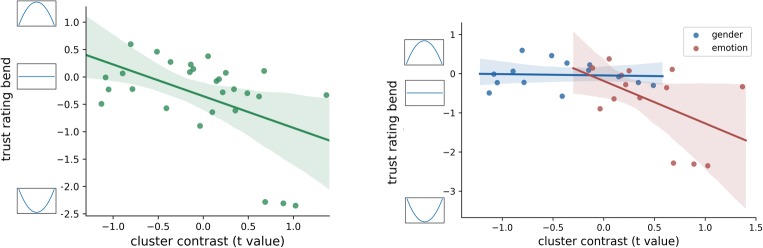
Correlation between single-subject interaction cluster t-value contrast (mix vs pure) and the divergence from a linear trend observed in the behavioral trust ratings. Y-axis values close to zero indicate a linear form of trust ratings, below zero indicate a U-shaped form, and above zero indicate a reverse Ushaped form. Panel A: Correlation for all subjects. Panel B: Correlations performed separately for each experimental categorization condition

As shown in Fig. 6A, single-subject *t*-values were negatively correlated with the divergence from a linear trend observed in behavioral trust ratings, *r*(27) = −0.495, *p* = 0.007. This means that the more negatively a participant evaluated mixed expression (greater “bend” of the rating line), the larger the ERP’s amplitude difference was between mixed and pure expressions. Interestingly, a similar analysis of a correlation between cluster contrast and the “bend” of categorization RTs did not reveal any significant differences.

One possible concern about the correlation of the whole group of points coming from two conditions is that it can be driven by the condition difference (Fig. 6B). To alleviate this concern, we conducted the correlations separately for each condition and found the following for the emotion condition: *r* = −0.50, *p* = 0.069, and for the gender condition: *r* = −0.05, *p* = 0.857. Although the correlation within the emotion condition is not nominally significant, note that it is based on fewer participants than the whole group correlation. The key point is that the correlation on the whole group is not (or at least not entirely) driven by the between-group difference. The presence of a correlation trend in the emotion condition and no correlation in the gender condition is in line with our reasoning; the interaction cluster, interpreted as a correlate of categorization conflict, should relate to the trust ratings only in the emotion condition, because in the gender condition, there is no categorization conflict. Overall, these findings are consistent with the interpretation of the LPP component as related to decision conflict, which negatively influences evaluative ratings.

## Discussion

In this research, we investigated the psychological and neural mechanisms underlying the link between facial features, fluency, ambiguity, and social evaluation. These results advance our theoretical understanding of how facial ambiguity is processed and how it translates into social judgments. This is particularly important in the domain of facial expressions, given that in real life people base consequential social judgments on expressive features of faces (Todorov, 2017) and that such features often are inherently ambiguous (Hassin, Aviezer, & Bentin, 2013).

The current study confirmed on the behavioral level that ambiguity (such as mixed facial expression) increases categorization time and decreases trustworthiness ratings. Notably, as theoretically predicted, these effects occurred only when the categorization task was related to the ambiguous stimulus dimension, i.e., emotions, but not the unambiguous dimension, i.e., gender. These results are consistent with previous research with blends of emotions (Olszanowski et al., 2018; Winkielman, Olszanowski & Gola, 2015) and also with blends across dimensions of gender (Owen et al., 2016), ethnicity (Halberstadt et al., 2014), humanness (Carr et al., 2017a), and facial identity (Carr et al., 2017b; Halberstadt, Pecher, Zeelenberg, Wai, & Winkielman, 2013).

The key novel contribution of the study was the ability to use EEG measures to better identify the mechanisms involved in the processing and evaluation of pure and ambiguous emotional faces and to examine their links to ratings of impressions. We will now review our key findings.

In the early time window (100 ms), we found a negative cluster at the fronto-central sites that, regardless of categorization condition, reflected a stronger response to pure expressions (angry and happy) compared with mixed expressions (middle frames). This N1 effect might reflect the same brain process as the P1, representing two projections of the same dipolar source (Joyce & Rossion, 2005) where more negative amplitude for pure expressions at N1 presumably translates into more positive amplitude at the P1 (Rossion et al., 1999a, b). We interpret this result as suggesting that the early brain responses reflect a simple match of basic, low-level features, with pure expressions offering a better template match than mixed expressions (Holmes et al., 2003).

In the later time window, we obtained two distinct LPP effects. One LPP effect occurred over central-parietal sites in the windows of 728 to 880 ms. This effect was independent of the categorization task and was greater to pure over mixed stimuli. Interestingly, a similar effect was obtained by Duval et al. (2013). These authors had participants view facial morphs ranging from happy-to-neutral and angry-to-neutral (without any categorization task). They found that the LPP over central-parietal sites followed the morph intensity such that faces with more intense affect (less neutral morph) elicited larger LPPs compared with faces depicting less intense affect (more neutral morph). Duval et al. (2013) interpreted their results as demonstrating the sensitivity of the LPP to more socially important facial expressions. We interpret our central-parietal LPP effect similarly in that it reflects processes for which mixed (morphed) expressions are less important or salient than pure expressions. It is also interesting to note again that “pure” faces represent a match to the stored template, which presumably generates higher familiarity of stimuli that match the prototypical emotion categories (Rugg et al. 1998). Finally, it is worth noting that work on affective pictures in general (facial and nonfacial) suggests that the LPP is sensitive to stronger affective content of emotional stimuli, such as IAPS pictures (Cuthbert et al., 2000).

More interestingly, we also obtained another, very different LPP effect over frontal midline sites in a slightly earlier time window (560-660 ms). Specifically, there was a significant interaction between categorization condition and stimulus ambiguity, reflecting a *larger* (more positive) response to ambiguous stimuli, but only in the emotion-categorization condition. One suggestion on how to interpret this effect comes from a recent review of LPP findings with affective stimuli (Hajcak & Foti, in press). They suggest that the amplitude of LPP depends on the salience of the processed stimulus, such that top-down manipulations that enhance stimulus significance tend to increase LPP amplitude. Applying this to our results, one possible interpretation is that the emotion categorization condition (which requires a binary decision) increases the task significance of the mixed, ambiguous faces (which accounts for longer decision RTs). To resolve ambiguity, participants may even attempt to create an additional third emotion category from the blend of happy and anger expressions (see Watson & Stanton, 2017 for discussion of how people create new emotions from blends). This interpretation could be tested in future studies by examining if the frontal midline LPP component tracks participants’ belief that they found such a “third” expression and its potential significance. Still, it is worth noting that “objectively” our mixed stimuli did not contain evidence for a different “third” expression (Fig. 1B), at least as determined by computer expressions recognition toolbox (CERT, Bartlett et al., 2008).

On our preferred interpretation, the frontal midline LPP reflects the mental effort of resolving ambiguity. This interpretation is in line with findings previously reported with face stimuli (Debruille, Brodeur, & Hess, 2011; Sun et al. 2017; Willadsen-Jensen & Ito, 2006) but also with non-face stimuli (Engel, Fries, & Singer, 2001). Presumably, the categorization task activated specific representations of prototypical emotional expressions (happy and angry), which highlight conflict between specific features in the case of mixed displays. Importantly, our study is unique in linking this LPP component to the decrease in trustworthiness ratings for those mixed, ambiguous displays. Our findings are consistent with Sun and his collaborators (2017) who found greater LPP to ambiguous facial expressions but only when participants had to categorize the stimuli on the ambiguous dimension. Aided by fMRI localization data, they interpreted these findings as reflecting cognitive effort generated by sources related to dACC, dmPFC, and IFG (see also Nomura et al., 2003). Spatial location of our LPP component is consistent with such interpretations.

More generally, our LPP findings indicate the complex nature of this ERP component. The current understanding is that LPPs are generated and modulated by an extensive brain network composed of both cortical and subcortical structures associated with perceptual, cognitive, and cognitive processing, and that LPP is not only sensitive to valence but also depends on the task context determining the salience of specific stimuli (Gable, Adams, & Proudfit, 2015; Hajcak & Foti, in press; Liu, Huang, McGinnis-Deweese, Keil, & Ding, 2012).

Overall, our results provide a better understanding of the interplay between facial features, processing effort, categorization, and social judgments. We showed that neural and behavioral responses depend on (i) objective characteristics of the stimuli (such as degree of emotion) and (ii) external task demands (such as categorization on the emotional dimension). Obtained results suggest that early neural responses are primarily dependent on basic stimulus features and can be impervious to top-down categorization set. This also is true for some late neural responses (i.e., central-parietal LPP), which appear to track parameters related to familiarity, prototypicality, and/or emotional intensity of the stimulus category, regardless of the categorization task. Critically, the more frontally generated LPP depends on a top-down processing set. This can highlight the categorization conflict for mixed stimuli and generate disambiguation effort, leading to an enhanced neural response. This frontal-midline LPP effect also relates to behavior, in the form of lower trustworthiness ratings. These findings are in line with our previous work using facial EMG where emotionally ambiguous expressions elicited lower zygomaticus (smiling) reactions compared with “pure” expressions, but only relatively late, approximately 2,000 ms after stimulus onset (Winkielman, Olszanowski & Gola, 2015). Of course, the timing of brain ERP measures clearly differs from peripheral EMG measures, which track activity of facial muscles that can reflect some slow processes (e.g., induction of an affective response).

Future research should extend these findings and address their limitations. For example, it is important to know whether the current results are limited to facial emotion stimuli or extend to ethnicity-ambiguous, gender-ambiguous, or identity-ambiguous faces. Given previous research showing preferences for unambiguous patterns, they also could extend to nonfacial stimuli (Vogel, Carr, Davis, & Winkielman, 2018; Winkielman et al., 2006). It also would be important to combine EEG and EMG measures to examine relations between brain (LPP) responses and peripheral affective (facial EMG) responses and thus to understand better the mechanisms generating the aversive nature of ambiguity. The addition of precise physiological measures of affect would help to address the limitation of our study regarding the interpretation of the LPP components as it relates to stimulus significance, intensity, effort, prototypicality, familiarity, and ambiguity/uncertainty. From the perspective of the social implications of these results, it is important to explore how the history of individual experiences with a variety of different faces and facial expressions can modify what is perceived as prototypical or ambiguous (Dotsch, Hassin, & Todorov, 2016; Principe & Langlois, 2012). Furthermore, it is useful to know how changes in top-down social categorization and labeling of “in-between” cases may prevent (or enhance) negative reactions to faces crossing emotional and social categories (Halberstadt & Winkielman, 2013). Lastly, it is valuable to test whether these neural and rating measures help us to predict actual trust-related behavior in real social interactions.

## Electronic supplementary material

ESM 1 (DOCX 15 kb)
