# Supplementary material for: Ambiguous at the second sight: Mixed facial expressions trigger late electrophysiological responses linked to lower social impressions
Source: Cogn Affect Behav Neurosci. 2020 Mar 12;20(2):441–54. doi: 10.3758/s13415-020-00778-5 (PMC7105445; doi:10.3758/s13415-020-00778-5)
Supplement: Supplementary file 1 — (DOCX 15 kb) [file 13415_2020_778_MOESM1_ESM.docx]

*Supplementary Materials for: “Ambiguous at the second sight: Mixed facial expressions trigger late electrophysiological responses linked to lower social impressions.”*

***General explanation of cluster-based analyses***

All EEG analyses were conducted using non-parametric cluster-based permutation tests (Maris and Oostenveld, 2007). The cluster based permutation method allows researchers to analyze the differences between conditions in the EEG signal using data from multiple electrodes and timepoints and apply relevant corrections for multiple comparisons. Thus, it allows one to look at the neural processes on a larger, data-driven scale rather than investigate only the averages of apriori selected electrodes and time windows.

The cluster-based permutation test consists of three steps. First, a selected statistical test is performed for each point in the chosen search space (for example every electrode - timepoint combination) giving rise to as many statistical results as there are search-space points (for example, the number of channels x the number of timepoints). In the second step, these statistical tests are thresholded according to a test statistic value (e.g., t-value) or p-value (e.g., p < 0.05). This produces a subset of points where the test result passed the specified threshold. These threshold-passing points are then grouped according to adjacency in the search space, giving rise to clusters where each cluster is continuous in the search space. In other words, in the electrode-timesamples search space, each cluster consists of spatio-temporally continuous threshold-passing points. Each cluster is then described by a cluster statistic - a single value summarizing the strength of all the statistical tests that form the cluster. This is most commonly the sum of cluster-forming statistical tests (for example, the sum of t-values).

In the last step, to assign p-values to observed cluster statistics, a null distribution of the maximum cluster statistic is simulated by permuting the data. Each permutation is a random assignment of the data to the compared conditions. For example, subject-level ERPs are randomly assigned to group labels or within-subject ERPs are randomly assigned to within-subject condition labels. For each permutation, the process of performing statistical test on search space points, thresholding and forming clusters is repeated, but only the highest cluster statistic is retained. The result of performing multiple (usually hundreds or thousands) permutations is a null distribution of the maximum cluster statistic to which the observed cluster statistics obtained in the actual, non-permuted contrast are compared.

The probability of an observed cluster statistic given the null distribution (cluster p-value) is the proportion of values in the null distribution that are higher than that of the observed cluster. Cluster p-values are then used to reject the hypothesis of equality between the conditions (or more precisely: interchangeability of the conditions) with a pre-defined alpha criterion. Because each cluster is aggregated into a single value and all observed clusters are compared to a null distribution of the maximum cluster statistic, this approach corrects for multiple comparisons. If the cluster statistic allows for both negative and positive values (like t statistic), the clusters are formed by threshold-passing statistics of the same polarity and the null distribution is constructed for both positive and negative cluster values separately.

Maris, E., & Oostenveld, R. (2007). Nonparametric statistical testing of EEG-and MEG-data. *Journal of Neuroscience Methods*, *164*(1), 177-190
